# Relationships between apolipoprotein E and insulin resistance in patients with obstructive sleep apnoea: a large-scale cross-sectional study

**DOI:** 10.1186/s12986-024-00816-w

**Published:** 2024-07-02

**Authors:** ZhiCheng Wei, Ling Tian, Huajun Xu, Chenyang Li, Kejia Wu, Huaming Zhu, Jian Guan, Yafeng Yu, Di Qian, Xinyi Li

**Affiliations:** 1 Department of Otorhinolaryngology Head and Neck Surgery, Shanghai Sixth People’s Hospital Affiliated to Shanghai Jiao Tong University School of Medicine, Shanghai Key Laboratory of Sleep Disordered Breathing, Otorhinolaryngology Institute of Shanghai JiaoTong University, Shanghai, 200233 China; 2 Donghai County Maternal and Child Health Hospital, No. 80, Shanxi Road, Niushan Street, Donghai County, Lianyungang City, 200233 JiangSu China; 3 Department of Otorhinolaryngology Head and Neck Surgery, First affiliated hospital of Soochow University, Suzhou City, 25006 Jiangsu China; 4 Department of otolaryngology, People’s Hospital of Longhua, 38 Jinglong construction Road, Longhua district, Shenzhen, China

**Keywords:** Apolipoprotein E, Insulin resistance, Mediation analysis, Non-linear, Obstructive sleep apnoea

## Abstract

**Background:**

Obstructive sleep apnoea (OSA) is commonly associated with insulin resistance (IR) and dyslipidaemia. Apolipoprotein E (APOE) plays important roles in lipid metabolism. The study aimed to disentangle the multifactorial relationships between IR and APOE based on a large-scale population with OSA.

**Methods:**

A total of 5,591 participants who underwent polysomnography for OSA diagnosis were finally enrolled. We collected anthropometric, fasting biochemical and polysomnographic data for each participant. Linear regression analysis was performed to evaluate the relationships between APOE, IR, and sleep breathing-related parameters. Logistic regression, restricted cubic spline (RCS) and mediation analyses were used to explore relationships between APOE and IR in patients with OSA.

**Results:**

Increasing OSA severity was associated with greater obesity, more obvious dyslipidaemia, and higher levels of APOE and IR. APOE was positively correlated with the apnoea-hypopnoea index (AHI), oxygen desaturation index (ODI) and microarousal index (MAI) even after adjusting for age, sex, body mass index, and smoking and drinking levels (β = 0.107, β = 0.102, β = 0.075, respectively, all *P* < 0.001). The risks of IR increased from the first to fourth quartiles of APOE (odds ratio (OR) = 1.695, 95% CI: 1.425–2.017; OR = 2.371, 95% confidence interval (CI): 2.009–2.816; OR = 3.392, 95% CI: 2.853–4.032, all *P* < 0.001) after adjustments. RCS analysis indicated non-linear and dose response relationships between APOE, AHI, ODI, MAI and insulin resistance. Mediation analyses showed that HOMA-IR explained 9.1% and 10% of the association between AHI, ODI and APOE. The same trends were observed in men, but not in women.

**Conclusions:**

This study showed that APOE is a risk factor for IR; moreover, IR acts as a mediator between OSA and APOE in men. APOE, IR, and OSA showed non-linear and multistage relationships. Taken together, these observations revealed the complex relationships of metabolic disorders in patients with OSA, which could lead to the development of new treatment modalities and a deeper understanding of the systemic impact of OSA.

**Supplementary Information:**

The online version contains supplementary material available at 10.1186/s12986-024-00816-w.

## Introduction

Obstructive sleep apnoea (OSA) is a prevalent sleep disorder characterised by chronic intermittent hypoxia (CIH) and fragmented sleep, which affects 23.4% of women and 49.7% of men [1]. It is strongly linked to type 2 diabetes (T2DM), insulin resistance (IR), obesity, hypertension, dyslipidaemia and cardiovascular diseases [2–4]. However, the research on the associations between these OSA complications, particularly between IR and lipid metabolism, is limited.

Studies have identified a high prevalence of IR and dyslipidaemia in patients with OSA [5, 6]. It has been shown that pro-atherogenic lipoprotein abnormalities correlated with IR rather than with OSA severity or the degree of hypoxia in nondiabetic, overweight/obese OSA adults [7]. Our previous study found that the APOB/APOA-I ratio was a risk factor for IR in OSA, and IR may serve as a mediator in OSA-related dyslipidaemia [8]. Furthermore, IR tends to be more strongly associated with lipid profiles in OSA patients compared to non-OSA individuals [9]. Apolipoprotein E (APOE), a key lipid transport protein that binds to various lipid species including cholesterol, phospholipids, and triglycerides (TGs) in lipoprotein particles [10, 11], is closely associated with adipocyte and body fat mass [10]. Elevated plasma levels of APOE have been linked to increased risks of mortality from all causes, cardiovascular disease, and cancer [12]. Research on the APOE-IR relationship has largely focused on insulin and cardiovascular disease mechanisms in APOE knockout mice [13], as well as the effect of APOE gene variations on brain insulin metabolism in human cognitive impairment [14, 15].

Allelic variants of the APOE gene modify cellular and tissue lipid metabolism [16, 17], suggesting that targeting APOE-associated metabolic pathways could influence OSA-related phenotypes. Over the past decade, studies have explored the relationship between OSA risk and specific APOE gene haplotypes (ε2/ε3/ε4; defined by the loci rs429358 and rs7412). A meta-analysis found no significant association between APOE ε2/ε3/ε4 variants and OSA susceptibility [18]. while some evidence suggests the APOE ε4 allele could increase OSA risk by 1.41 times [19]. Additionally, possessing at least one APOE ε2 allele was associated with a 9.37-fold greater risk of OSA [20]. Nevertheless, research on serum APOE and OSA is limited, though a non-linear, multistage dose–response relationship between serum APOE levels and OSA severity has been reported [21]. The apnoea–hypopnoea index (AHI) in rapid eye movement (REM) sleep has been linked to a 2.7-fold increase in APOE levels after adjusting for confounding factors [22]. Studies examining APOE genotype and OSA relationships have yielded varied results.

To date, the relationship between serum APOE and IR patients with OSA remains unclear. In this large, cross-sectional study, we investigated the linear and non-linear relationships between APOE, IR, and OSA.

## Methods

### Participants

This study was conducted as part of ongoing research focusing on individuals with suspected OSA who visited our hospital’s sleep centre for a standard whole-night polysomnography (PSG). The research aimed to explore the relationships between OSA, metabolic disorders, and genetic predispositions. From 2007 to 2016, a total of 6,433 participants from our hospital’s sleep centre were included. Exclusion criteria were as follows: age < 18 years; regular use of lipid-lowering drugs; history of OSA treatment; severe liver, renal, or heart failure; cancer, psychiatric diseases, or pregnancy; and/or other sleep-related or respiratory diseases (i.e. chronic obstructive pulmonary disease, asthma, restless leg syndrome, narcolepsy, or insomnia). Patients with missing IR and APOE data were also excluded. Finally, 5591 patients were included in the study. A flow chart of participant selection is presented in Figure S1.

This study was performed in accordance with the Declaration of Helsinki and the protocol was approved by the Ethics Committee of Shanghai Jiao Tong University Affiliated Sixth People’s Hospital. All participants provided written informed consent.

### Anthropometric and biochemical measurements

Anthropometric measurements were conducted by trained technicians on participants wearing light clothing and no shoes, following standardised protocols. The procedures were performed in accordance with standard protocols, and the mean values of paired measurements were used for analysis. Height and weight were measured using a calibrated electronic scale, and the readings were recorded. Body mass index (BMI) was calculated as weight (kg)/height^2^ (m^2^). Waist circumference (WC) was measured at the middle of the lowest costal margin and the iliac crest during expiration using a flexible tape measure. Hip circumference (HC) was measured at the widest part of the buttocks with the measurer standing to the side. The waist/hip ratio was calculated as WC/HC. Blood pressure and diastolic blood pressure were measured in triplicate after a resting interval of at least 10 min, with the participant seated and the upper arm at heart level, using an automated electronic device (Model HEM-752 Fuzzy; Omron Co., Kyoto, Japan); the mean values of three readings were used for analysis.

A fasting blood sample was obtained the morning after polysomnographic monitoring. Fasting blood glucose (FBG), total cholesterol (TC), TG, HDL-C, LDL-C, APOA, APOB, and APOE were measured using an autoanalyser (H-7600; Hitachi, Tokyo, Japan) in the Shanghai Sixth People’s Hospital laboratory. Serum fasting insulin was measured by immunoassay. Homeostasis model assessment of IR (HOMA-IR) was calculated as fasting insulin (µIU/mL) × FBG (mmol/L)/22.5. HOMA-IR ≥ 2.5 was considered indicative of IR [23].

### Polysomnographic evaluation and sleep assessment

Overnight standard PSG (Alice 4 or 5; Respironics Inc., Pittsburgh, PA, USA) was performed, consisting of electroencephalography, electrooculography, chin electromyography, and electrocardiography to assess sleep status, nasal/oral airflow, and thoracic/abdominal movement; finger pulse oximetry was performed to assess respiratory events. The PSG readings were analysed using the software supplied with the Alice device, then manually checked by experienced sleep technicians in accordance with the updated 2012 American Academic Sleep Medicine criteria (data collected before 2012 were rescored) [24]. Apnoea was defined as reduction in airflow from baseline of ≥ 90% lasting for at least 10 s; hypopnoea was defined as a ≥ 30% reduction in airflow accompanied by a decrease in SpO2 of ≥ 3% or an arousal. OSA severity was determined by the AHI, with AHI < 5, < 15, < 30, and ≥ 30 per hour defined as non-OSA, mild, moderate, and severe OSA, respectively. The oxygen desaturation index (ODI) was calculated as the number of episodes of oxygen desaturation ≥ 3% per hour during sleep. The microarousal index was calculated as the number of arousals per hour of sleep.

### Statistical analysis

All statistical analyses were performed using SPSS version 22.0 (IBM Corp., Armonk, NY, USA). The Kolmogorov–Smirnov test was used to determine data distribution normality. Normally distributed and skewed continuous data are presented as means ± standard deviations or medians and interquartile ranges (25–75%), respectively. Categorical data are expressed as numbers (percentages). *P*-values for linear trends were calculated using the polynomial linear trend test for continuous variables and the linear-by-linear association test for dichotomous variables. Additionally, multivariate logistic regression models were used to examine the associations between APOE and IR with or without adjustments for age, sex, and BMI, using the lowest APOE level as the reference. IR (represented by HOMA-IR) was evaluated as an intermediate variable mediating the relationships between the characteristics of OSA (AHI, ODI, and MAI) and APOE in mediation analysis. Two-sided *P*-values < 0.05 were considered indicative of statistical significance.

Restricted cubic spline (RCS) analysis, a powerful statistical method for investigating dose–response and non-linear multistage relationships in epidemiology and clinical studies, was used [25, 26]. RCS was analysed to elucidate the dose–effect relationship between APOE and OSA/IR using MATLAB 8.0 (MathWorks Inc., Natick, MA, USA).

## Results

### Baseline characteristics

We enrolled 5,591 eligible participants, including 716 with non-OSA, 302 with mild OSA, 1,146 with moderate OSA and 3,427 with severe OSA. Table 1 presents the baseline anthropometric indices, sleep-breath-related parameters and biochemical characteristics. Participants with OSA had greater obesity and greater hypoxia. TC, TG, LDL-C, APOB, APOE, FBG and fasting insulin increased with increasing OSA severity. The rates of IR in non, mild, moderate and severe OSA groups were 32.3%, 41.1%, 51.8% and 64.2%, respectively. Non-smoker percentages in the non-OSA, moderate and severe OSA categories were 76.7%, 66.6%, 79.6% and 76.4%, respectively. Similarly, the percentages of non-drinkers in these groups were 51.7%, 68.9%, 56.0% and 54.4%, respectively.

**Table 1:** Baseline of the overall population by the severity of OSA

Characteristics	Non-OSA (*N* = 716)	Mild (*N* = 302)	Moderate (*N* = 1146)	Severe (*N* = 3427)	*P* for trend
**Demographics**					
Age, years	35(29–44)	33(29–36)	44(35–54)	43(35–54)	< 0.001
Male(%)	716(100%)	302(100%)	915(79.8%)	3039(88.7%)	< 0.001
Height, cm	1.72(1.7–1.76)	1.73(1.7–1.78)	1.7(1.65–1.75)	1.72(1.68–1.75)	< 0.001
Weight, Kg	71.25(65–80)	75(70–82)	75(68–84)	81(74–90)	< 0.001
BMI, Kg/m^2^	24.22(22.38–26.17)	25.31(23.38–27.15)	25.95(24.07–28.08)	27.72(25.51–30.07)	< 0.001
SBP, mmHg	120(113–130)	120(120-124.5)	125(116–135)	128(120–138)	< 0.001
DBP, mmHg	78(71–83)	80(79.5–80)	80(72–87)	81(76–90)	< 0.001
NC, cm	38(36.5–40)	39(37–41)	39(37–41)	41(39–43)	< 0.001
WC, cm	90(84–95)	91.5(86.25-97)	94(89–100)	99(93–105)	< 0.001
HC, cm	98(94–102)	100(96–103)	100(96–104)	103(99–107)	< 0.001
WHR	0.91(0.88–0.95)	0.92(0.89–0.95)	0.94(0.91–0.98)	0.96(0.93-1.00)	< 0.001
**Obstructive sleep apnea**					
ESS	5(1–10)	7(4–11)	7(3–11)	10(5–14)	< 0.001
AHI	2.10(0.8–3.4)	10.45(8.7–12.8)	21.80(18.3–25.7)	57.40(44.7–70.2)	< 0.001
Minimum SaO2	92(89–94)	87(84–90)	82(78–87)	71(62–79)	< 0.001
ODI	2.30(1.1–3.9)	10.60(7.80–13.6)	21.95(17.3–27.5)	57.70(43.7–72.1)	< 0.001
Mean SaO2	96(95.3–97)	96(95–97)	95(94–96)	93(90-94.4)	< 0.001
MAI	14.50(10.1–22.6)	18.60(12.6–27.0)	21.10(13.5–31.3)	35.90(20.7–54.6)	< 0.001
**Biochemistry assays**					
TC, mmol/l	4.41(3.87–5.03)	4.54(4.06–5.13)	4.72(4.15–5.41)	4.83(4.27–5.44)	< 0.001
TG, mmol/l	1.32(0.89–1.94)	1.35(0.91–2.06)	1.60(1.12–2.33)	1.76(1.26–2.57)	< 0.001
HDL-C, mmol/l	1.03(0.91–1.19)	1.06(0.91–1.19)	1.02(0.89–1.19)	1.00(0.88–1.14)	< 0.001
LDL-C, mmol/l	2.69(2.24–3.21)	2.84(2.41–3.36)	2.95(2.45–3.47)	3.30(2.51–3.55)	< 0.001
APOA, g/L	1.03(0.93–1.15)	1.03(0.92–1.14)	1.04(0.93–1.17)	1.05(0.94–1.17)	0.043
APOB, g/L	0.78(0.66–0.89)	0.82(0.69–0.94)	0.84(0.73–0.97)	0.87(0.76-1.00)	< 0.001
APOE, mg/dl	3.81(3.18–4.71)	3.88(3.34–4.91)	4.27(3.52–5.25)	4.48(3.61–5.67)	< 0.001
Lipoprotein a, mg/dl	7.84(4.10–16.90)	7.81(4.00-16.31)	7.35(3.70-15.15)	7.20(3.70–14.90)	0.069
Glucose (mmol/L)	5.09(4.78–5.44)	5.02(4.75–5.36)	5.25(4.91–5.69)	5.37(5.00-5.96)	< 0.001
Insulin (uU/ml)	8.08(5.55–11.68)	9.73(6.62–13.43)	10.68(7.30-15.41)	12.80(8.72–18.80)	< 0.001
HOMA-IR	1.85(1.18–2.77)	2.23(1.50–3.21)	2.60(1.69–3.88)	3.15(2.05–4.89)	< 0.001
**Medical history**					
Non-smoker, N(%)	549(76.7%)	201(66.6%)	912(79.6%)	2618(76.4%)	< 0.001
Non-drinker, N(%)	370(51.7%)	208(68.9%)	642(56.0%)	1843(54.4%)	< 0.001
IR(%)	231(32.3%)	124(41.1%)	594(51.8%)	2199(64.2%)	< 0.001

Skewed data are presented as the median (IQR), and categorical data as the number (percentage). Differences in the baseline characteristics among the four groups were examined using the Nonparametric test for continuous variables and the linear-by-linear association test for dichotomous variables. BMI, body mass index; NC, neck circumference; WC, waist circumference; HC, hip circumference; WHR, waist/hip ratio; SBP, systolic blood pressure; DBP, diastolic blood pressure; TC, total cholesterol; TG, triglyceride; HDL-C, high-density lipoprotein cholesterol; LDL-C, low-density lipoprotein; cholesterol; APOA, apolipoprotein A; APOB, apolipoprotein B; APOE, apolipoprotein E; ESS, Epworth Sleepiness Scale; AHI, apnoea–hypopnea index; SaO2, oxygen saturation; ODI, oxygen desaturation index; MAI, micro-arousal index. HOMA-IR, homeostasis model assessment for insulin resistance; IR, insulin resistance

### Associations between APOE and risk of IR

The associations between lipid levels, HOMA-IR and sleep-breathing related indictors are shown in Table 2. HOMA-IR was positively correlated with AHI, ODI, and APOE levels both before (β = 0.188, 0.191, and 0.172; all *P* < 0.001) and after adjustments for age, sex, and BMI (β = 0.067, 0.065, and 0.128; all *P* < 0.001), and remained significant after further adjustment for smoking and drinking levels (β = 0.066, 0.065, and 0.129; all *P* < 0.001). HOMA-IR was positively correlated with MAI without adjustments (β = 0.067; *P* < 0.001), but it showed no association after adjustments for age, sex, and BMI (β = 0.026; *P* = 0.069). APOE was associated with AHI, ODI, and MAI even after adjustments for age, sex, and BMI (β = 0.107, 0.102, and 0.071; all *P* < 0.001), and this remained consistent after further adjustments (β = 0.107, β = 0.102, β = 0.075, respectively, all *P* < 0.001).

**Table 2:** linear regressions APOE, HOMA-IR, and sleep-breath related parameters

	AHI		ODI		MAI		HOMA-IR	
	ß	*P*	ß	*P*	ß	*P*	ß	*P*
**Model 1**								
TC, mmol/l	0.156	< 0.001	0.146	< 0.001	0.091	< 0.001	0.113	< 0.001
TG, mmol/l	0.147	< 0.001	0.147	< 0.001	0.126	< 0.001	0.196	< 0.001
HDL-C, mmol/l	-0.070	< 0.001	-0.061	< 0.001	-0.035	0.012	-0.119	< 0.001
LDL-C, mmol/l	0.126	< 0.001	0.119	< 0.001	0.101	< 0.001	0.045	0.001
APOA, g/L	0.013	0.345	0.009	0.509	-0.029	0.042	-0.019	0.166
APOB, g/L	0.176	< 0.001	0.160	< 0.001	0.085	< 0.001	0.124	< 0.001
APOE, mg/dl	0.173	< 0.001	0.173	< 0.001	0.098	< 0.001	0.172	< 0.001
Lipoprotein a, mg/dl	-0.049	< 0.001	-0.050	< 0.001	-0.040	0.004	-0.036	< 0.01
HOMA-IR	0.188	< 0.001	0.191	< 0.001	0.084	< 0.001	-	-
**Model 2**								
TC, mmol/l	0.096	< 0.001	0.085	< 0.001	0.067	< 0.001	0.076	< 0.001
TG, mmol/l	0.086	< 0.001	0.084	< 0.001	0.096	< 0.001	0.157	< 0.001
HDL-C, mmol/l	-0.018	0.162	-0.010	0.434	0.004	0.759	-0.086	< 0.001
LDL-C, mmol/l	0.081	< 0.001	0.074	< 0.001	0.078	< 0.001	0.015	0.247
APOA, g/L	0.030	0.015	0.025	0.042	-0.009	0.530	-0.003	0.831
APOB, g/L	0.105	< 0.001	0.088	< 0.001	0051	< 0.001	0.079	< 0.001
APOE, mg/dl	0.107	< 0.001	0.102	< 0.001	0.071	< 0.001	0.128	< 0.001
Lipoprotein a, mg/dl	-0.034	< 0.001	-0.032	< 0.01	-0.030	0.028	-0.021	0.112
HOMA-IR	0.067	< 0.001	0.065	< 0.001	0.026	0.069	-	-
**Model 3**								
TC, mmol/l	0.097	< 0.001	0.086	< 0.001	0.059	< 0.001	0.076	< 0.001
TG, mmol/l	0.086	< 0.001	0.084	< 0.001	0.092	< 0.001	0.157	< 0.001
HDL-C, mmol/l	-0.018	0.146	-0.010	0.428	-0.009	0.528	-0.089	< 0.001
LDL-C, mmol/l	0.081	< 0.001	0.076	< 0.001	0.066	< 0.001	0.015	0.268
APOA, g/L	0.030	0.016	0.025	0.044	-0.001	0.958	-0.003	0.833
APOB, g/L	0.105	< 0.001	0.088	< 0.001	0.054	< 0.001	0.079	< 0.001
APOE, mg/dl	0.107	< 0.001	0.102	< 0.001	0.075	< 0.001	0.129	< 0.001
Lipoprotein a, mg/dl	-0.034	0.005	-0.032	0.008	-0.033	0.015	-0.021	0.107
HOMA-IR	0.066	< 0.001	0.065	< 0.001	0.024	0.096	-	-

Model 1: No adjustment

Model 2: Adjusted for age, sex, BMI

Model 3: Adjusted for age, sex, BMI, smoking status, drinking status

We divided APOE into four quartiles, with the first quartile regarded as the reference (Table 3). The risk of IR increased as the APOE quartile increased from the first to the fourth in model 1 (OR = 1.534, 95% confidence interval (CI): 1.317–1.786; OR = 2.415, 95% CI: 2.071–2.816; OR = 4.382, 95% CI: 3.725–5.156, all *P* < 0.001) and with adjustments for age, sex, and BMI in model 2 (OR = 1.425, 95% CI: 1.211–1.677; OR = 2.071, 95% CI: 1.758–2.440; OR = 3.475, 95% CI: 2.924–4.129, all *P* < 0.001), and with further adjustments for age, sex, BMI, smoking and drinking in model 3 (OR = 1.695, 95% CI: 1.425–2.017; OR = 2.371, 95% CI: 2.009–2.816; OR = 3.392, 95% CI: 2.853–4.032, all *P* < 0.001). The same trends were observed for men, whereas compared with the lowest quartile, the second quartile was not associated with an increased risk of IR in women.

**Table 3:** Risk of insulin resistance according to tertiles of APOE in OSA subjects

APOE	Model 1	*p* value	Model 2	*p* value	Model 3	*p* value
**Total**						
Q1	1	-	1	-	1	-
Q2	1.534(1.317–1.786)	< 0.001	1.425(1.211–1.677)	< 0.001	1.695(1.425–2.017)	< 0.001
Q3	2.415(2.071–2.816)	< 0.001	2.071(1.758–2.440)	< 0.001	2.371(2.009–2.816)	< 0.001
Q4	4.382(3.725–5.156)	< 0.001	3.475(2.924–4.129)	< 0.001	3.392(2.853–4.032)	< 0.001
**Men**						
Q1	1	-	1	-	1	-
Q2	1.485(1.269–1.737)	< 0.001	1.409(1.188–1.672)	< 0.001	1.738(1.462–2.065)	< 0.001
Q3	2.407(2.052–2.825)	< 0.001	2.107(1.711–2.506)	< 0.001	2.841(2.399–3.365)	< 0.001
Q4	4.199(3.544–4.975)	< 0.001	3.460(2.883–4.152)	< 0.001	4.206(3.550–4.984)	< 0.001
**Women**						
Q1	1	-	1	-	1	-
Q2	1.554(0.945–2.557)	0.082	1.688(0.979–2.908)	0.059	2.055(1.309–3.227)	0.002
Q3	2.040(1.273–3.269)	0.003	1.999(1.184–3.377)	0.010	2.764(1.716–4.451)	< 0.001
Q4	4.139(2.525–6.785)	< 0.001	4.082(2.349–7.094)	< 0.001	4.601(2.778–7.621)	< 0.001

APOE and risks of insulin resistance were examined using multivariable logistic regression models

In total subjects

Model 1: No adjustment

Model 2: Adjusted for age, sex, BMI

Model 3: Adjusted for age, sex, BMI, smoking status, drinking status

### Mediation analysis

**Fig. 1:**
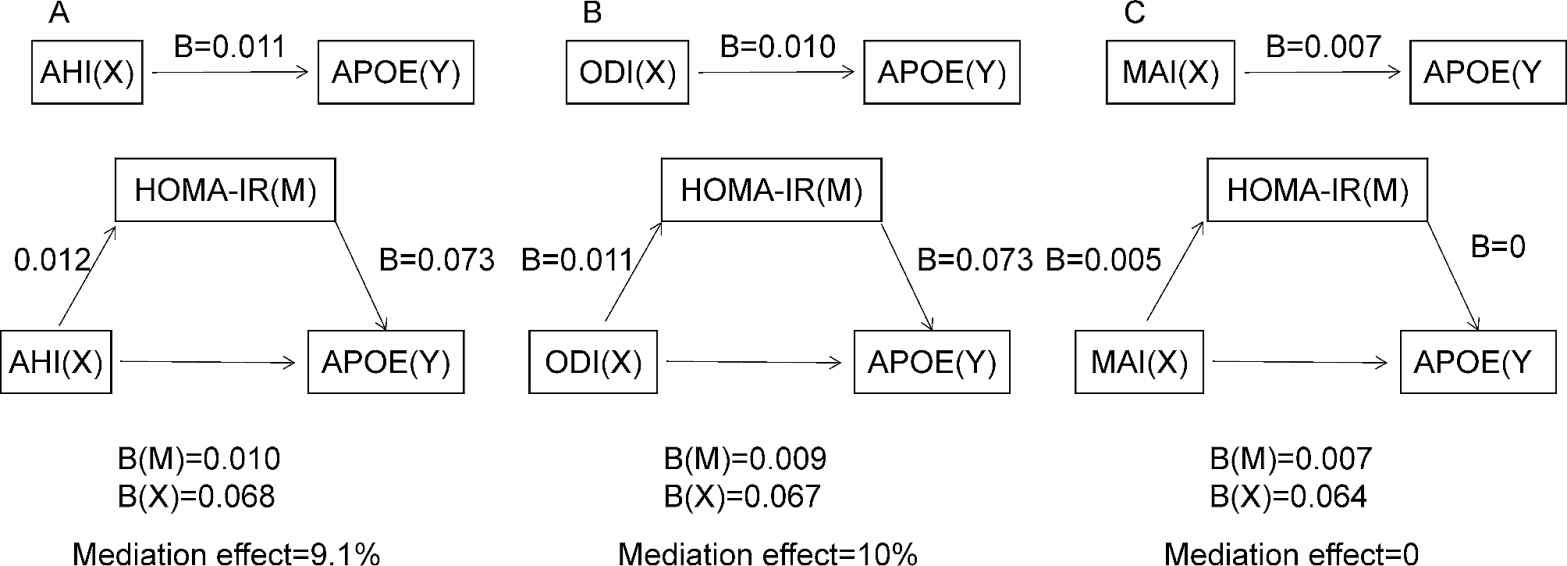
Path diagram showing how HOMA-IR mediates the relationships between OSA measures and APOE. All analyses were adjusted for age, sex, and BMI. (**A**) HOMA-IR mediated the association between AHI and APOE. (**B**) HOMA-IR mediated the association between ODI and APOE. (**C**) HOMA-IR did not mediate the association between MAI and APOE X, main predictor; Y, APOE; M, HOMA-IR (mediator); B, unstandardised coefficient

We performed mediation analyses to explore potential causal relationships between OSA, HOMA-IR and APOE. The detailed steps of these analyses are shown in Tables S1, S2, and S3. The results of the mediation analyses estimated that HOMA-IR accounts for 9.1% and 10% of the association between AHI, ODI and APOE after adjusting for age, sex, BMI (Fig. 1A, B). HOMA-IR did not mediate the association between MAI and APOE (Fig. 1C).

### RCS analysis

**Fig. 2:**
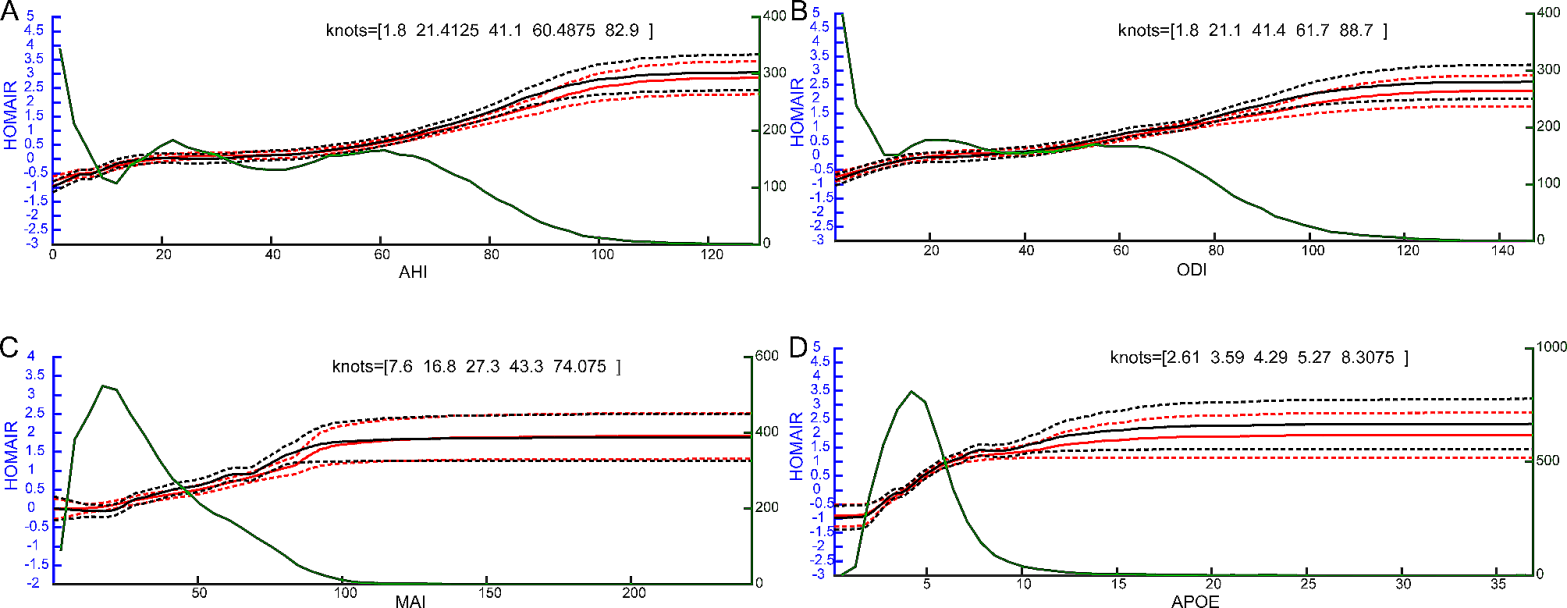
Restricted cubic spline regression of the multistage correlation patterns of AHI, ODI, MAI, and APOE with risk of IR. Left y-axis represents log odds of IR. Right y-axis represents number of patients. The x-axis represents continuous values of AHI (Fig. **2A**), ODI (Fig. **2B**), MAI (Fig. **2C**), or APOE (Fig. **2D**). Population number for each OSA severity measure unit is indicated by the green line. Red line, without adjustment; black line, adjusted for age, sex, and BMI. Solid lines indicate odds ratios, and dashed lines indicate 95% confidence intervals

We regarded APOE as a continuous variable (Fig. 2, x-axis), and the log odds of IR (Fig. 2, y-axis) were analysed as two categorical variables to delineate the dose-effect relationships between AHI, ODI, MAI APOE and the risk of IR. The analysis revealed that the risk of IR did not increase linearly with AHI, ODI, MAI, or APOE levels. Initially, the log odds curve showed slow changes or fluctuations at early stages of OSA (AHI scores of 5–20), with turning points in the curves then indicating a rapid change in association with AHI, ODI, MAI and APOE. Knots were calculated for AHI (1.8, 21.4, 41.1, 60.5, and 82.9) (Fig. 2A), ODI (1.8, 21.1, 41.4, 61.7, and 88.7) (Fig. 2B), MAI (7.6, 16.8, 27.3, 43.3, and 74.1) (Fig. 2C), and APOE (2.61, 3.59, 4.29, 5.27, and 8.31) (Fig. 2D). For detailed description, the change in the risk of IR exhibited a rapid rise at 2.61 knots of APOE, followed by a plateau between 3.59 and 4.29 knots, and then a gradual increase with further rises in APOE. There is a declining growth trend at knots 8.31. Similar trends were also observed in other panels when the data were stratified by sex (Figure S2).

## Discussion

This study was performed to examine comprehensively the relationships between IR, APOE and OSA using current large-scale sampling and strict data acquisition methods. The results showed that APOE had significant positive relationships with AHI, ODI, MAI and IR after adjusting for age, sex and BMI. Furthermore, the study revealed a non-linear dose–response relationship between APOE levels and the risk of IR, particularly in males with OSA. Additionally, HOMA-IR may mediate the association between OSA and APOE. These findings indicated that there are complex relationships between lipid and glucose metabolism in OSA patients, suggesting that early interventions targeting one aspect of OSA may beneficially influence other associated conditions.

The complex interplay between IR and APOE has been implicated in the mechanisms of cognitive impairment [14, 27]. APOE may regulate insulin sensitivity by downregulating the basal activity of the insulin receptor signalosome [28]. Furthermore, cerebrovascular glucose load and IR exhibit an APOE ε4-dependent response [29]. A 5-year follow-up study demonstrated that middle-aged APOE ε4 allele carriers with IR are at increased risk for late-life Aβ accumulation [30]. Regarding metabolic derangements, ε4 allele carriers exhibited significantly higher plasma concentrations of TC, LDL-C and APOB compared to ε2 carriers and the ε3/ε3 genotype [31]. Previous studies have demonstrated that APOE contributes to excessive fat accumulation and IR in ApoE^−/−^ mice [32]. a Conversely, in obese individuals with diabetes, IR may trigger an increase in very low-density lipoprotein (VLDL)-induced APOE production [33]. These findings suggest a complex interplay between APOE and IR, with direct and indirect effects.

OSA, a component of metabolic syndrome [34], is independently associated with the underlying causes of dyslipidaemia and IR [5, 35]. However, analyses investigating the interrelationships between OSA, serum APOE and IR remain limited. Our study identified elevated APOE levels as a risk factor for IR in OSA patients. This finding contrasts with the Framingham Offspring Study, which did not establish an association between APOE polymorphisms and IR [36]. CIH, a defining characteristic of OSA, induces IR and exacerbates atherosclerosis in ApoE^−/−^ mice [37]. Hypoxia-inducible factors contribute to this process by increasing reactive oxygen species (ROS) production, leading to CIH-induced IR [38]. Therefore, we suggest that CIH conditions may exacerbate the interaction between APOE and IR.

Caveolin-1, which is crucial for insulin receptor-mediated signaling and the development of IR, was found to colocalise with APOE at the plasma membrane of adipocytes during adipogenesis [39, 40]. The expression of caveolin-1 is induced under hypoxic conditions [41], and its downregulation has been associated with increased levels of HIF-1α [42]. Therefore, we assumed that caveolin-1 might play a mediating role in the interaction between IR and APOE in CIH. The results indirectly supported our finding that IR is a potential mediator between OSA and APOE. Additionally, we identified a non-linear multistage relationship between APOE levels, OSA-related traits, and IR. The mechanisms underlying the observed plateau and turning points in this relationship remain unclear. It is recognised that not all associations are linear, and they may depend on the exposure dose [25]. The plateau and turning points observed might represent protective adaptations that lead to remodelling of lipid and glucose homeostasis in patients with CIH in the context of OSA.

This study had some limitations. First, because it was a large-scale observational study, causation could not be explored; additional longitudinal or multicentre randomised clinical studies are required. Both genetic and environmental factors play important roles in the aetiology of dyslipidaemia and OSA; however, physical exercise, lifestyle, and genetic factors were not considered in this study. Additionally, since the participants were hospital patients and the prevalence of OSA is higher in men than in women, selections bias may have affected the findings related to sex. Finally, the mechanisms of IR and APOE remain unclear; they should be explored in future studies. Despite these limitations, this was the first investigation of the relationships between APOE and risk of IR in patients with OSA reported thus far. The complexity of these relationships implies that OSA and its related complications are multifactorial and complicated.

## Conclusions

The results from this study reveal non-linear and dose-dependent relationships between IR, APOE, and OSA. APOE was found to increase the risk of IR in patients with OSA, with IR potentially mediating the relationship between OSA and APOE. These results contribute to a better understanding of the complex nature of OSA and provide a solid foundation for personalised treatments of metabolic comorbidities associated with OSA.

### Electronic supplementary material

Below is the link to the electronic supplementary material.

Supplementary Material 1

Supplementary Material 2

Supplementary Material 3

## Data Availability

All data during this study can provide if needed.

## Abbreviations

OSA: Obstructive sleep apnoea
APOE: Apolipoprotein E
BMI: Body mass index
NC: Neck circumference
WC: Waist circumference
HC: Hip circumference
WHR: Waist/hip ratio
HOMA-IR: Homeostasis model assessment for insulin resistance
SBP: Systolic blood pressure
DBP: Diastolic blood pressure
TC: Total cholesterol
TG: Triglyceride
HDL-C: High-density lipoprotein cholesterol
LDL-C: Low-density lipoprotein; cholesterol
APOA: Apolipoprotein A
APOB: Apolipoprotein B
OR: Odds ratio
CI: Confidence interval
