# Supplementary material for: Relationships between apolipoprotein E and insulin resistance in patients with obstructive sleep apnoea: a large-scale cross-sectional study
Source: Nutr Metab (Lond). 2024 Jul 2;21:40. doi: 10.1186/s12986-024-00816-w (PMC11221003; doi:10.1186/s12986-024-00816-w)
Supplement: Supplementary file 1 — Supplementary Material 1 [file 12986_2024_816_MOESM1_ESM.docx]

|  | coefficient | se | P |
| --- | --- | --- | --- |
| Y=APOE  X=AHI  +adjustments | 0.011 | 0.001 | 0.000000 |
| Y=HOMA-IR  (mediator)  X=AHI  +adjustments | 0.012 | 0.002 | 0.000000 |
| Y= APOE  X=HOMA-IR  (mediator)  +adjustments | 0.073 | 0.007 | 0.000000 |
| Y=APOE  X=AHI  M=HOMA-IR  (mediator)  +adjustments | 0.010  0.068 | 0.001  0.007 | 0.000000  0.000000 |
| Indirect effect | 0.011-0.010=0.001 |  |  |
| Sobel approach | 0.012*0.068=0.000816 | 0.00015985 | 3.3×10^-7^ |

**Table 1S Mediation analysis by multivariate Linear regression analysis, testing associations with sleep apnea and APOE mediated by HOMA-IR following steps below running outcome variable (APOE), main predictor is AHI, and mediator (HOMA-IR)**

Adjustments include: age, sex, BMI;AHI, apnea hypopnea index; BMI, body mass index; APOE, apolipoprotein E.

Mediation effect=9.1%

**Table 2S Mediation analysis by multivariate Linear regression analysis, testing associations with sleep apnea and APOE mediated by HOMA-IR following steps below running outcome variable (APOE), main predictor is ODI, and mediator (HOMA-IR)**

|  | coefficient | se | P |
| --- | --- | --- | --- |
| Y=APOE  X=ODI  +adjustments | 0.010 | 0.001 | 0.000000 |
| Y=HOMA-IR  (mediator)  X=ODI  +adjustments | 0.011 | 0.002 | 0.000000 |
| Y= APOE  X=HOMA-IR  (mediator)  +adjustments | 0.073 | 0.007 | 0.000000 |
| Y=APOE  X=ODI  M=HOMA-IR  (mediator)  +adjustments | 0.009  0.067 | 0.001  0.007 | 0.000000  0.000000 |
| Indirect effect | 0.010-0.009=0.001 |  |  |
| Sobel approach | 0.011*0.067=0.000737 | 0.00015455 | 0.00000185 |

Adjustments include: age, sex, BMI;AHI, apnea hypopnea index; BMI, body mass index; APOE, apolipoprotein E.

Mediation effect=10%

**Table 3S Mediation analysis by multivariate Linear regression analysis, testing associations with sleep apnea and APOE mediated by HOMA-IR following steps below running outcome variable (APOE), main predictor is MAI, and mediator (HOMA-IR**

|  | coefficient | se | P |
| --- | --- | --- | --- |
| Y=APOE  X=MAI  +adjustments | 0.007 | 0.001 | 0.000000 |
| Y=HOMA-IR  (mediator)  X=MAI  +adjustments | 0.005 | 0.003 | **0.069189** |
| Y= APOE  X=HOMA-IR  (mediator)  +adjustments | 0.073 | 0.007 | 0.000000 |
| Y=APOE  X=MAI  M=HOMA-IR  (mediator)  +adjustments | 0.007  0.064 | 0.001  0.007 | 0.000000  0.000000 |
| Indirect effect | 0.007-0.007=0 |  |  |
| Sobel approach | 0.005*0.064=0.00032 | 0.00019516 | 0.10107872 |

Adjustments include: age, sex, BMI;AHI, apnea hypopnea index; BMI, body mass index; APOE, apolipoprotein E.

Mediation effect=0
