# Supplementary figures and images for: Relationships between apolipoprotein E and insulin resistance in patients with obstructive sleep apnoea: a large-scale cross-sectional study
Source: Nutr Metab (Lond). 2024 Jul 2;21:40. doi: 10.1186/s12986-024-00816-w (PMC11221003; doi:10.1186/s12986-024-00816-w)

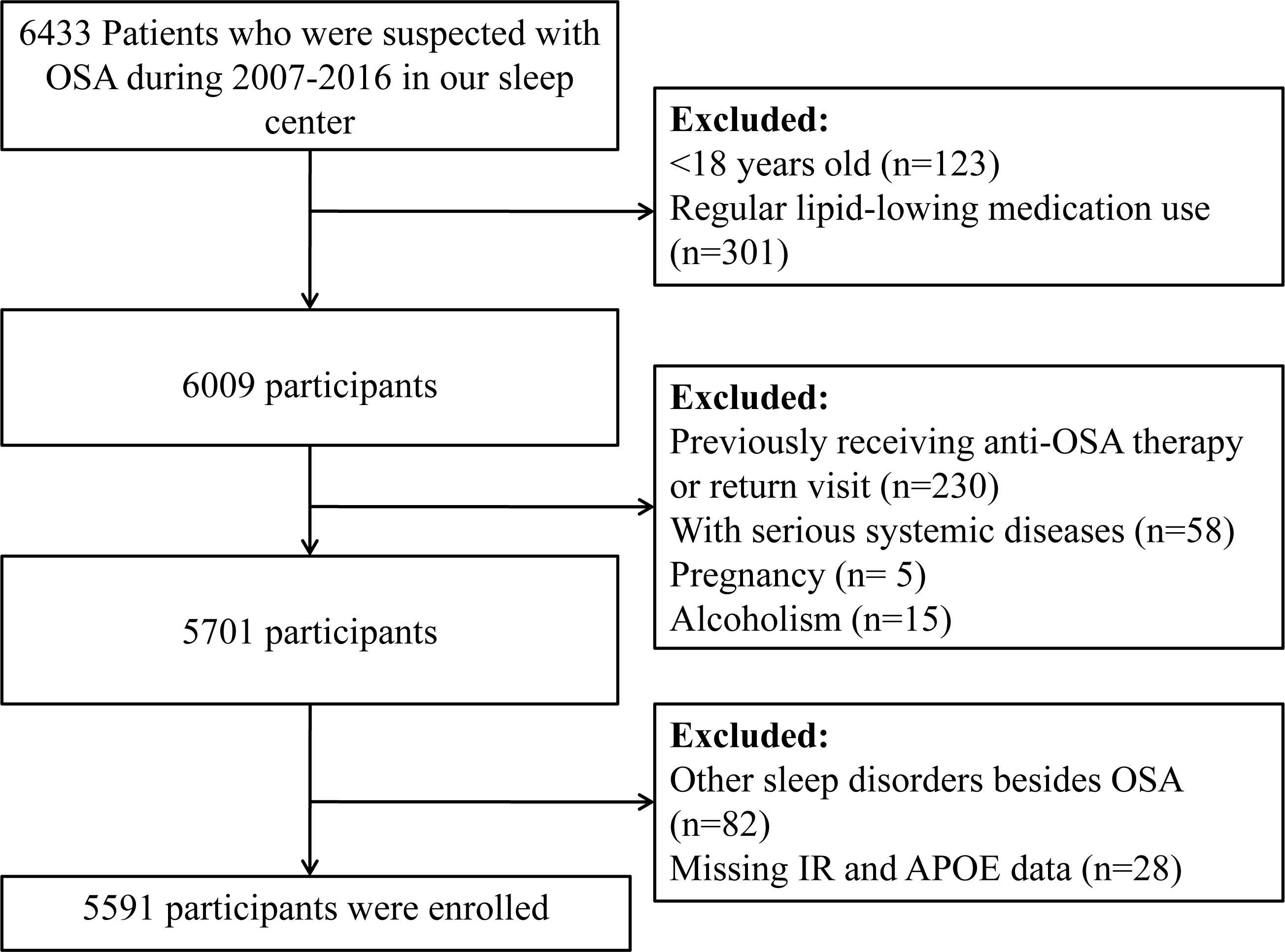

Supplement: Supplementary file 2 — Supplementary Material 2 [file 12986_2024_816_MOESM2_ESM.png]
